# Linguistical and psychometric validation of the MIND Youth Questionnaire (My-Q) in Swedish

**DOI:** 10.1186/s41687-026-01148-4

**Published:** 2026-07-14

**Authors:** Anna Lena Brorsson, David Rudilla, Max Kleijberg, Maartje deWit, Elena Lundberg, Anna Lindholm-Olinder

**Affiliations:** 1 Department of Women’s and Children’s Health, Karolinska Institutet, Stockholm, Sweden; 2 Astrid Lindgren Children’s Hospital, Karolinska University Hospital, Stockholm, Sweden; 3 Pulmonology, Air Liquide Healthcare, Hospital Universitario de la Princesa, Madrid, Spain; 4 Department of Neurobiology, Care Sciences and Society, Karolinska Institutet, Stockholm, Sweden; 5 Department of Health Sciences, Innovation and Design, Mälardalen University, Eskilstuna, Sweden; 6 Medical Psychology, Vrije Universiteit Amsterdam, Amsterdam, Netherlands; 7 Department of Pediatrics, Institution of Clinical Science, Umeå University, Umeå, Sweden; 8 Department of Clinical Science and Education, Karolinska Institutet, Stockholm, Sweden; 9 Sachs Children and Youth Hospital, Stockholm South General Hospital, Stockholm, Sweden

**Keywords:** Questionnaire, Type 1 diabetes, Validation, Youth

## Abstract

**Background:**

The Mind Youth Questionnaire is a multidimensional health-related quality-of-life questionnaire designed for pediatric diabetes care and exists in Dutch, English, and Spanish.

**Methods:**

The aim of this study was to linguistically and psychometrically validate the Mind Youth Questionnaire (My-Q) for Swedish-speaking youths with type 1 diabetes. The linguistic process of the Dutch version followed ISPOR´s guidelines. For face validity, youths with type 1 diabetes and diabetes nurses were interviewed. Three stigma items were included. The final Swedish My-Q consists of 38 items. A total of 166 youths (10–19 years) completed the My-Q and Pediatric Quality of Life Inventory diabetes module. A 2nd -order confirmatory factor analysis was conducted using the nine-factor Dutch and five-factor Spanish versions.

**Results:**

The 2nd -order nine-factor solution (social impact, parents, diabetes control perceptions, responsibilities, worries, treatment satisfaction, body image and eating behavior, stigma, and mood) showed adequate fit: χ2(551) = 828.36 (*p* < .01), Comparative Fit Index (CFI) = 0.90, Root Mean Square Error of Approximation (RMSEA) = 0.05 [0.05,0.06], Standardized Root Mean Square Residual (SRMR) = 0.08. The nine-factor solution and grouping of the items followed a clear rationale. The reliability coefficients of the total scale were adequate, α = 0.86, Ω = 0.90, and Ω for all the factors ranged from 0.49 to 0.88. The relationships between My-Q factors and sociodemographic variables revealed that boys had better health-related quality of life than girls. Younger youths reported better health-related quality of life in terms of body image and eating behavior (*p*=.005). Concurrent validity was confirmed, as all the factors were positively related to all the Pediatric Quality of Life Inventory diabetes module factors. A negative correlation was found between the level of glycated hemoglobin and the My-Q score (*r*=-.258, *p* < .001).

**Conclusion:**

The Swedish My-Q has adequate psychometric properties and can be used in research and for routine psychosocial assessment.

**Supplementary Information:**

The online version contains supplementary material available at 10.1186/s41687-026-01148-4.

## Background

Approximately 1.8 million children and adolescents (0–19 years) have type 1 diabetes (T1D) worldwide [1]. Sweden is one of the countries with the highest incidence, with 45 per 100 000 inhabitants and nearly 9,000 children (0–18 years) living with T1D [2]. Living with T1D involves substantial self-care responsibilities, which include continuous independent decision-making regarding insulin dosage on the basis of glucose values, carbohydrate intake, and degree of physical activity. Optimal glucose control is needed to reduce the risk of acute and long-term complications [3].

The importance of assessing person-reported outcomes (PROs) clinically in children and adolescents with T1D is emphasized internationally and nationally [4, 5]. The use of person-reported outcome measures (PROMs) in a clinical context can facilitate a person’s ability to report symptoms and reflect on them, which, in conversations between a young person, legal guardians (when applicable), and healthcare professionals may, to a greater extent, result in shared decision-making and person-centered care [6]. A structured assessment of PRO, followed by a respectful conversation about problems, has been appreciated by young people and is effective in improving mental health and satisfaction with care [7, 8]. Clinically, the use of questionnaires on a regular basis provides the opportunity to detect common problems in young people with diabetes, especially depressive symptoms and eating disorders, problems that warrant professional evaluation, and possible interventions [7].

The questionnaires used in Sweden to measure health-related quality of life (HRQoL) include the “disabled children’s quality-of-life measure” (DISABKIDS-36) [9] and the “Pediatric Quality of Life Inventory” (PedsQL 3.0) [10]. The Monitoring Individual Needs in Diabetes (MIND) Youth Questionnaire (MY-Q) is based on a consensus from health care professionals and young people with T1D who identified aspects that should be included, as well as a review of existing PROMs for the target group, with a focus on validity and clinical utility. The MY-Q is designed for use in routine care for children and adolescents with T1D and identifies PRO, as they experience it, on a physical, emotional, social, and mental level [11, 12]. Although PedsQL and DISABKIDS provide valuable data for research, their implementation in busy clinical settings can be challenging due to the complexity of score interpretation. The MY-Q seeks to bridge this gap by offering an actionable format; it utilizes a scoring algorithm that ‘flags’ specific high-risk responses, allowing clinicians to identify psychosocial needs and barriers to self-management in real-time without the need for extensive psychometric analysis during the visit. The MY-Q has been developed and psychometrically evaluated in children and adolescents with T1D in the Netherlands and has shown good validity and reliability [12]. A Spanish translation and validation of the MY-Q has been performed, showing adequate psychometric properties and conceptual and semantic equivalence with the original Dutch version [13].

## Methods

### Aim

The aim of the current study was to linguistically and psychometrically validate the MY-Q for Swedish-speaking youth with T1D.

### Design

This is a linguistic and psychometric validation of the Swedish MY-Q. The STROBE checklist was used when preparing the manuscript [14].

### Questionnaires

The original Dutch MY-Q consists of 32 items divided into seven domains: social impact, parents, diabetes control perceptions, responsibility, worries, treatment satisfaction, body image and eating behavior, and mood (emotional wellbeing) using the WHO-5 questionnaire. All the items use a 5- or 6-point Likert scale pertaining to frequency or intensity. It also includes an item about the general QoL of life (response scale of 1–10), an item about treatment barriers (the youth select items they consider from a list), and two open-ended items conclude the questionnaire, which asks about life events in the past six months and any topic(s) the teenager would like to discuss with the health care team [12]. The Spanish version consists of three domains (negative impact of diabetes, empowerment, and control of diabetes and worry), and the WHO-5 questionnaire measures emotional wellbeing [13].

In addition to the MY-Q, the HRQoL was measured for concurrent validity via the diabetes-specific module of the PedsQL (PedsQL 3.0). The PedsQL 3.0 consists of 28 items and five scales: (i) diabetes symptoms (11 items), (ii) treatment barriers (four items), (iii) treatment adherence (seven items), (iv) worry (three items), and (v) communication (three items). The PedsQL 3.0 uses a 5-point Likert scale (ranging from 0 = never to 4 = almost always). Items are reverse-scored and transformed to a 0–100 scale, with higher scores indicating better HRQoL [10].

### The linguistic process

**Fig. 1:**
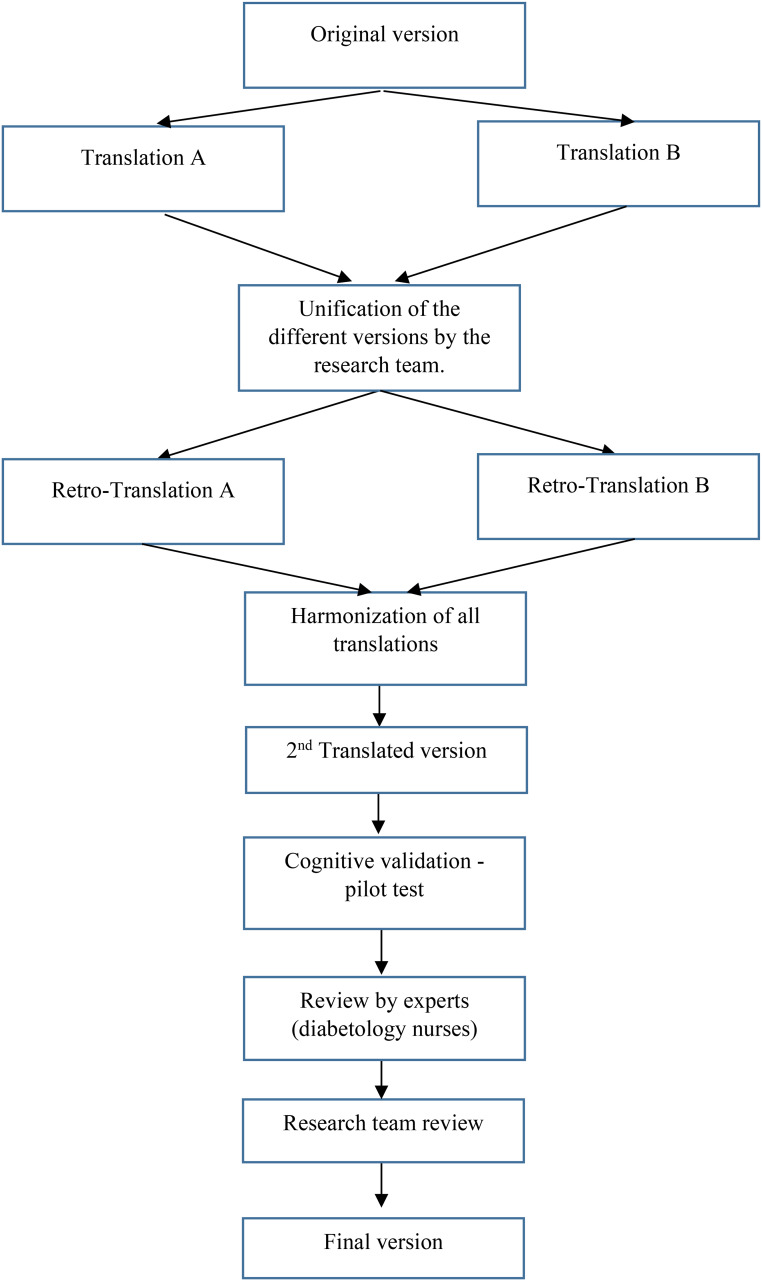
Phases of the cultural-linguistic adaptation process of the MY-Q to Swedish

The principles of good practices for the translation and cultural adaptation process described by the International Society for Pharmacoeconomics and Outcomes Research (ISPOR) were applied [15]. The linguistic process is illustrated in Fig. 1. Permission for translation and psychometric evaluation was obtained from the developer of the original MY-Q, the psychologist Maartje de Wit (The Netherlands), who is a co-author. Two native Dutch and Swedish speakers translated the original Dutch MY-Q into Swedish, after which two other native Dutch and Swedish speakers created a back-translation to Dutch. The two versions were reviewed and compared to the original questionnaire. In this process, a few small changes were made regarding the Swedish cultural and healthcare context. The face validity of the first draft of the Swedish MY-Q was performed through two digital meetings with a total of ten youths (10–18 years) with T1D and written comments from eight diabetes nurses. During the translation process, a matrix was used to collect information about the content, language, and any doubts that could be raised with questions related to comprehension and writing.

After discussion with the research team and suggestions from diabetes nurses during face validity, the need to include items about stigma was identified. The stigma items were selected by asking 23 people (12 diabetes nurses and 11 individuals with T1D) to identify the three most important stigmatizing statements in each domain of the Stigma Assessment Scale (DSAS-1) for adults with T1D (treated differently, blame and judgment, and identity concerns) [16]. Furthermore, one item within each domain of the DSAS-1, with the highest percentage of agree and strongly agree, was identified. These items are equivalent to the most important items identified by Swedish diabetes nurses and individuals with T1D. The selected items were translated into Swedish and culturally adapted. The research team finalized the questionnaire.

### Participants and settings in the validation study

Strategic selection was made when pediatric diabetes clinics were invited to participate. The goal was to include clinics from the whole country, which vary in size, and participants from urban and rural areas. Participants were recruited from nine pediatric diabetes clinics in Sweden in connection with a visit to the pediatric outpatient diabetes clinic. The inclusion criteria were age 10–19 years, a diagnosis of T1D, an understanding of the written Swedish language, and the ability to independently complete the questionnaire. For sample size calculation, we followed the recommendation of including a minimum sample size of 100–200 [17].

### Data collection

Data were collected at the included pediatric outpatient clinics between February 2023 and May 2024. The nurses were trained to administer the questionnaires. The participants answered the questionnaire digitally or on paper. The participants were given a personal code to complete the questionnaire. Sociodemographic data and diabetes-related variables, including age, sex, family structure, number and order of siblings, type of place of residence, type of treatment, and current glycated hemoglobin (HbA1c), were self-reported in the questionnaire.

### Data analysis

The data were analyzed via the SPSS version 28 and MPlus 8.1 statistical modeling programs [18, 19]. Descriptive statistics were calculated and, when applicable, are presented as the mean (SD). Descriptive and reliability statistics for the items and total score of the Swedish version of the MY-Q scale were calculated. These included the mean, SD, and minimum and maximum scores.

Given that the construct of the MY-Q (‘HRQoL in youth with T1D’) could load into certain underlying subconstructs or components for the study of the internal structure, two competitive 2nd -order factor analyses (CFAs) were tested with the two structures previously evaluated: the structure of the Dutch version [12] (composed of 9 factors including the new ‘Stigma’ factor) and the structure of the Spanish version [13] (composed of 5 factors including the new ‘Stigma’ factor). Owing to the ordinal nature of the data and their nonnormality, the estimation method used was weighted least square mean and variance adjusted (WLSMV). Model fit was assessed via the chi square statistic the Comparative Fit Index (CFI), with values of more than 0.90 (ideally 0.95) indicating good fit, and the Root Mean Square Error of Approximation (RMSEA), with values of 0.08 or less indicating excellent fit [20].

To estimate internal consistency, Cronbach’s alpha and McDonald’s omega were calculated. McDonald’s omega considers factor loadings, making it a more accurate and reliable indicator than Cronbach’s alpha is [21].

Furthermore, as a part of the validation, concurrent validity was assessed by relating the dimensions of the MY-Q to Hb1Ac and PedsQL 3.0 via Pearson correlations. We studied the relationships between the Swedish version of the MY-Q and sex, age, family structure, type of glucose monitoring, and type of treatment via *t* tests for independent samples, Pearson correlations, and analyses of variance (ANOVA).

## Results

### Linguistic results

The final Swedish version of the MY-Q consists of 38 items: one general question, 29 items in different domains of HRQoL, five items corresponding to the WHO-5 questionnaire, and three open-ended items. The MY-Q covers the following domains of general QoL: social life (friends, family, and school), diabetes management (worries, treatment barriers, self-efficacy and satisfaction, and problematic eating), and emotional well-being. The Swedish version includes the ‘stigma’ domain. Most questions use a 5-point Likert scale, indicating the frequency or intensity. Scores were transformed to a 100-point scale, with higher scores indicating better HRQoL.

### The validation

In this study, 166 patients from nine hospitals in Sweden participated. The mean age was 14.7 (2.1) years, 59% were girls, 44% lived in cities with fewer than 99 000 inhabitants, and 71.7% lived with both parents. The mean HbA1c level was 50.5 (9.0) mmol/mol (DCCT: 6.8 (0.8)%); 96.4% of the participants used a continuous glucose measurement (CGM), and 88.6% used an insulin pump (continuous subcutaneous insulin infusion, CSII) (Table 1).

**Table 1:** Demographic and clinical characteristics of the participants in the validation study

		Mean	Standard deviation
Age		14.7 [10–19]	2.1
Hb1Ac, mmol/mol DCCT-values, %		50.5 6.8	9.0 0.8
		**N**	**%**
Gender	Girl	98	59.0
	Boy	67	40.4
	Other	1	0.6
City size	Big city (more than 500.000 people)	54	32
	Medium-sized city (100.000-500.000 people)	40	24
	Small town (20.000- 99.999 people)	36	22
	Sparsely populated (less than 20.000 people)	36	22
Family structure	Living with both parents	119	71.7
	Alternating living with parents	27	16.3
	Living with mother	15	9.0
	Living with father	1	0.6
	Other	4	2.4
Number of siblings	0	19	11.4
	1	85	51.2
	2	45	27.1
	3	17	10.2
	No siblings	21	12.7
Order among siblings	1st	59	35.5
	2nd	67	40.4
	3rd	19	11.4
Glucose monitoring	Fingerstick for glucose measurement	6	3.6
	Continuous glucose measurement (CGM)	138	83.1
	Both	22	13.3
Treatment	Continuous Subcutaneous Insulin Infusion (CSII)	147	88.6
	Multiple daily injections (MDI)	19	11.4

To study the internal structure of the Swedish version of the MY-Q, a 2nd -order CFA including five-factor (Spanish version) [13] and the nine-factor (Dutch version) [12] solutions were tested. The overall fit was the most adequate for the 2nd -order nine-factor solution (Table 2). This model showed adequate fit: *χ*^*2*^(551) = 828.36 (*p* < .01), CFI = 0.90, RMSEA = 0.05 [0.05,0.06], Standardized Root Mean Square Residual (SRMR) = 0.08.

**Table 2:** Confirmatory factor analysis models’ overall fit

Model	χ^2^	d.f.	*p*	CFI	TLI	RMSEA [90%CI]	SRMR
5-correlated factors (Spanish) – 2nd Order	871.838	555	< 0.0001	0.88	0.88	0.059 [0.051, 0.66]	0.08
*9-correlated factors (Dutch) – 2nd Order*	*828.363*	*551*	*< 0.0001*	*0.90*	*0.90*	*0.054 [0.047*,* 0.063]*	*0.08*

Notes: Italics for the model retained

**Fig. 2:**
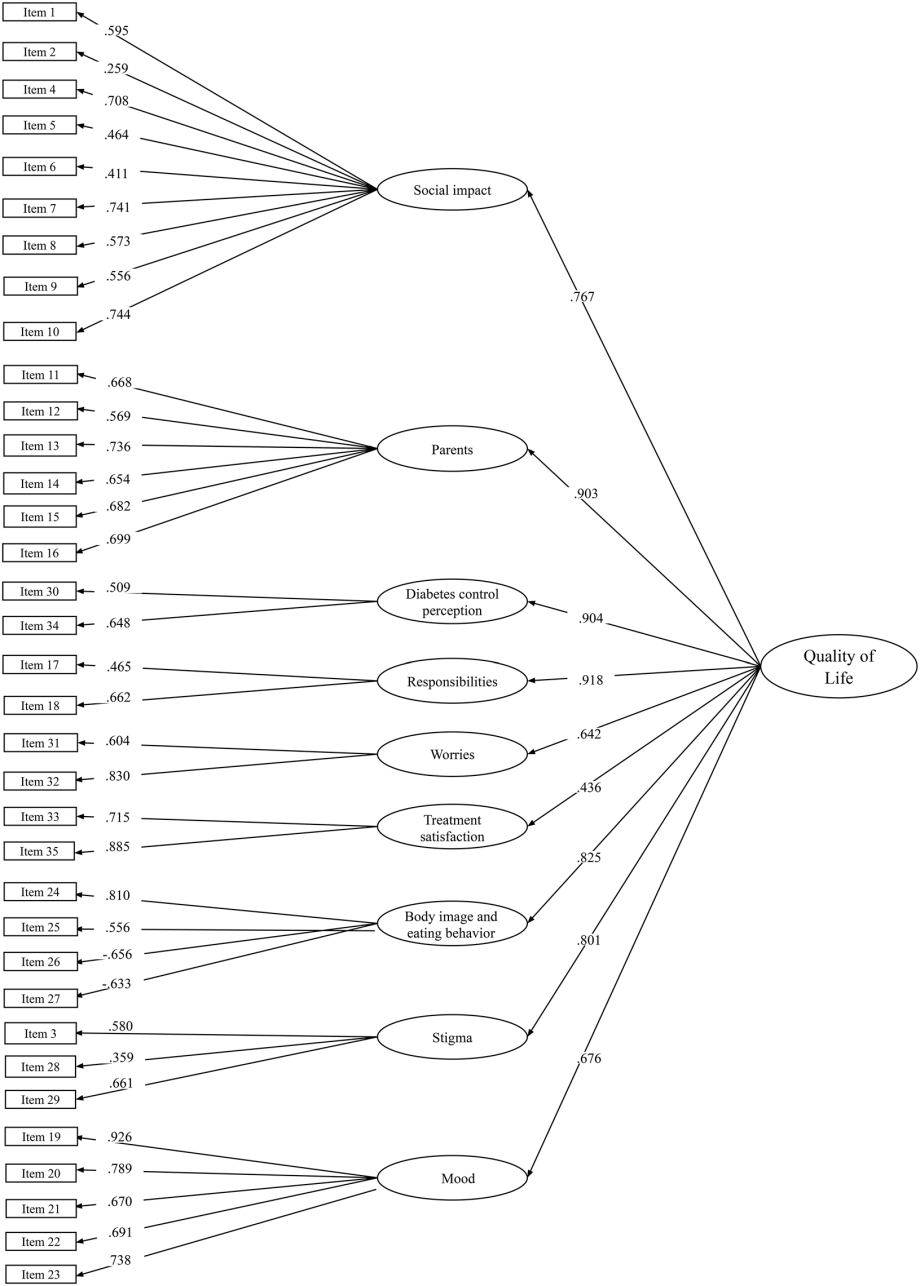
Analytical fit of the confirmatory factor analysis

The analytical fit of the 2nd -order nine-factor model is presented in Table 3. Table 4 shows the factors social impact, parents, diabetes control perceptions, responsibilities, worry, treatment satisfaction, body image and eating behavior, and mood explained by the same items as in the original Dutch structure. Furthermore, the stigma factor included items added to the Swedish model. Only Item 2 showed some peculiarities in its behavior. Although this item presented a low factor loading (0.26), its content was judged as relevant to measure the construct in addition to being statistically significant. Therefore, the items were grouped into nine factors that loaded on a 2nd -order construct (Table 4). All the 1st -order factor saturations in the 2nd order are greater than 0.6 (Fig. 2).

**Table 3:** Factor loadings and descriptive statistics for the MY-Q items

Item	*λ*	M	SD	Min.	Max.
1	*0.595*	58.43	22.95	0	100
2	*0.259*	70.33	28.81	0	100
3	*0.580*	85.24	22.20	0	100
4	*0.708*	71.99	21.63	0	100
5	*0.464*	84.04	18.49	0	100
6	*0.411*	94.88	11.52	50	100
7	*0.741*	54.22	23.61	0	100
8	*0.573*	63.40	28.68	0	100
9	*0.556*	52.26	24.28	0	100
10	*0.744*	87.05	16.45	25	100
11	*0.668*	66.11	30.15	0	100
12	*0.569*	91.27	15.29	25	100
13	*0.736*	47.74	30.00	0	100
14	*0.654*	63.25	31.58	0	100
15	*0.682*	68.37	31.40	0	100
16	*0.699*	69.43	29.69	0	100
17	*0.465*	74.25	27.58	0	100
18	*0.662*	75.15	26.75	0	100
19	*0.926*	68.31	24.43	0	100
20	*0.789*	56.74	28.98	0	100
21	*0.670*	60.36	27.71	0	100
22	*0.691*	44.45	32.16	0	100
23	*0.739*	58.79	31.50	0	100
24	*0.810*	65.96	26.99	0	100
25	*0.556*	62.05	32.99	0	100
26	*0.656*	88.86	19.17	0	100
27	*0.633*	93.22	14.14	25	100
28	*0.359*	47.74	40.80	0	100
29	*0.661*	58.13	41.67	0	100
30	*0.509*	80.72	17.11	0	100
31	*0.604*	80.42	22.20	0	100
32	*0.830*	68.98	27.41	0	100
33	*0.715*	85.84	22.59	0	100
34	*0.648*	62.80	23.14	0	100
35	*0.885*	81.17	21.22	0	100

Notes: λ = f factor loading; M= mean; SD= standard deviation; Min = minimum score; Max = maximum score. The item scores are recoded (1 = 0, 2 = 25, 3 = 50, 4 = 75, and 5 = 100), and the total score is computed as the mean of the items, resulting in scores between 0 and 100

**Table 4:** Structure of the MY-Q in Swedish: items and factors

	Item	F1	F2	F3	F4	F5	F6	F7	F8	F9
1	Det är svårt att vara uppmärksam i klassen/på jobbet. It is difficult to pay attention in class/at work.	X								
2	Jag kan lita på att mina lärare/kollegor tar hand om min diabetes om de skulle behöva göra det. *I can trust my teachers/colleagues to take care of my diabetes if they need to.*	X								
3	Jag har blivit diskriminerad i skolan för att jag har typ 1 diabetes. *I have been discriminated against at school because I have type 1 diabetes.*								X	
4	Hur ofta hindrar din diabetes dig i ditt sociala liv, vänskaper och relationer? *How often does your diabetes hinder you in your social life*,* friendships and relationships?*	X								
5	Jag kommer bra överens med jämnåriga *I get along well with my peers*	X								
6	Andra barn mobbar mig *Other kids bully me*	X								
7	Hur ofta stör din diabetes din fritid? *How often does your diabetes interfere with your free time?*	X								
8	Hindrar din diabetes dig från att göra saker utan dina föräldrar (exempelvis fester, sova över, gå ut)? *Does your diabetes prevent you from doing things without your parents (e.g. parties*,* sleepovers*,* going out)?*	X								
9	Hur ofta stör din diabetes dina idrottsaktiviteter (t.ex. fotboll, gymnastik, gym)? *How often does your diabetes interfere with your sports activities (e.g. soccer*,* gymnastics*,* gym)?*	X								
10	Hur ofta hindrar din diabetes aktiviteter med familjen? *How often does your diabetes interfere with family activities?*	X								
11	Hur ofta har du känslan av att du på grund av din diabetes är en belastning för någon i familjen (exempelvis dina föräldrar/bonusförälder, syskon eller mor/farföräldrar)? *How often do you feel that because of your diabetes you are a burden on someone in the family (for example your parents/bonus parent*,* siblings or mother/grandparents)?*		X							
12	Hur ofta känner du att dina föräldrar… … ger dig tillräckligt med hjälp och stöd för att hantera din diabetes? *How often do you feel that your parents give you enough help and support to manage your diabetes?*		X							
13	Hur ofta känner du att dina föräldrar oroar sig för mycket över din diabetes? *How often do you feel that your parents worry too much about your diabetes?*		X							
14	Hur ofta känner du att dina föräldrar beter sig som att det är deras diabetes? *How often do you feel like your parents act like it is their diabetes?*		X							
15	Under den senaste månaden har mina föräldrar och jag varit oense om att komma ihåg att kontrollera glukosvärden/ta insulin. *For the past month*,* my parents and I have been arguing about remembering to check glucose levels/take insulin.*		X							
16	Under den senaste månaden har mina föräldrar och jag varit oense om måltider och mellanmål. *For the past month*,* my parents and I have been disagreeing about meals and snacks.*		X							
17	Hur ofta känner du att du måste göra för mycket själv av din diabetesbehandling? *How often do you feel that you have to do too much of your diabetes treatment yourself?*				X					
18	Hur ofta känner du att andra gör för mycket av din diabetesbehandling? *How often do you feel that others are doing too much of your diabetes care?*				X					
19	Har jag känt mig glad och på gott humör *Have I felt happy and in a good mood*									X
20	Har jag känt mig lugn och avslappnad *Have I felt calm and relaxed*									X
21	Har jag känt mig aktiv och full av energi *Have I felt active and full of energy*									X
22	Har jag känt mig pigg och utvilad när jag vaknade. *Have I felt refreshed and rested when I woke up.*									X
23	Var min vardag fylld av saker som intresserar mig *My everyday life was filled with things that interest me*									X
24	Jag är nöjd med hur jag ser ut *I am happy with the way I look*							X		
25	Jag försöker (på olika sätt) att påverka min vikt *I try (in different ways) to influence my weight*							X		
26	Hur ofta har du de senaste 14 dagarna hetsätit? *How often in the last 14 days have you binge eaten?*							X		
27	Hur ofta har du de senaste 14 dagarna med flit hoppat över insulin? *How often in the last 14 days have you deliberately skipped insulin?*							X		
28	Vissa dömer mig om jag äter söt mat eller dryck eftersom jag har typ 1-diabetes (t.ex. kakor, godis, läsk). *Some judge me if I eat sweet foods or drinks because I have type 1 diabetes (e.g. cookies*,* candy*,* soda).*								X	
29	Jag skäms över vad folk kan tycka om jag behöver hjälp vid ett lågt blodsocker. *I am ashamed of what people might think if I need help with low blood sugar.*								X	
30	Jag känner att jag har kontroll över min diabetes *I feel I have my diabetes under control*			X						
31	Hur ofta oroar du dig för att du plötsligt ska bi medvetslös/få ett väldigt lågt blodsocker? *How often do you worry that you will suddenly pass out/have very low blood sugar?*					X				
32	Hur ofta oroar du dig för att du ska få komplikationer av din diabetes? *How often do you worry about complications from your diabetes?*					X				
33	Är du nöjd med ditt diabetesteam? *Are you satisfied with your diabetes team?*						X			
34	Är du nöjd med dina glukosvärden? *Are you satisfied with your glucose values?*			X						
35	Är du nöjd med den diabetesbehandling som du har nu? *Are you satisfied with the diabetes treatment you have now?*						X			

Notes: F1 = Social Impact; F2 = Parents; F3 = Diabetes Control Perceptions; F4 = Responsibilities; F5 = Worries; F6 = Treatment satisfaction; F7 = Body image and eating behavior; F8 = Stigma; F9 = Mood

The reliability estimates were calculated by considering the internal structure. The reliability coefficients of the scale were adequate, with α = 0.86 and Ω = 0.90. For the different factors, the Ω values were as follows: ‘Social Impact’ 0.81, ‘Parents’ 0.83, ‘Diabetes control’ 0.50, ‘Responsibilities’ 0.49, ‘Worries’ 0.69, ‘Treatment satisfaction’ 0.78, ‘Body image and eating behavior’ 0.76, ‘Mood’ 0.88 and ‘Stigma’ 0.55.

The levels of diabetes-related HRQoL for all the items are shown in Table 3. For ‘Social Impact’ (F1), items with higher scores were 6 (94.88) and 10 (87.05); lower scores were observed for item 9 (52.26). For ‘Parents’ (F2), a higher score was given for item 12 (91.27), and lower scores were given for item 13 (47.74). The item with the highest score for ‘Diabetes Control Perceptions’ (F3) was Item 30 (80.72), and the lowest score was Item 34 (62.80). For ‘Responsibilities’ (F4), the items had almost identical scores. For ‘Worries’ (F5), higher scores were given for Item 31 (80.42), and the lowest score was given for Item 32 (68.98). For ‘Treatment satisfaction’ (F6), the items had almost identical scores. The highest score for ‘Body image and eating behavior’ (F7) was for Item 27 (93.22), and the lowest score was for Item 25 (62.05). For ‘Stigma’ (F8), the highest score was for Item 3 (85.24), and the lowest score was for Item 28 (47.74). Finally, ‘Mood’ (F9) had a higher score for Item 19 (68.31) and a lower score for Item 22 (44.45).

With respect to the factors themselves, the means ‘Social Impact’ (70.73), ‘Diabetes Control Perceptions’ (71.76), ‘Responsibilities’ (76.69), ‘Worries’ (74.69) and ‘Body image and eating behavior’ (77.52) had medium–high scores. ‘Treatment satisfaction’ presented the highest score of all factors (83.50). On the other hand, ‘Parents’ (67.69), ‘Stigma’ (63.70) and ‘Mood’ (57.73) presented medium scores.

The levels of diabetes-related HRQoL for different factors are shown in Table 5. Overall, the perception of diabetes-related HRQoL was 66.20 (11.12). All factors are above the score of 60, except ‘Mood’ (57.73 (22.80)). In terms of gender, boys reported a better HRQoL than girls did in terms of both the global HRQoL (70.35 (10.15) vs. 62.36 (10.74), p<.001) and all factors. The differences between boys and girls are significant in the following factors: ‘Social Impact’ (p=.005), ‘Parents’ (p<.001), ‘Diabetes Control Perceptions’ (p<.001), ‘Responsibilities’ (p=.037), ‘Body image and eating behavior’ (p<.001), ‘Worries’ (p=.003), ‘Stigma’ (p=.002) and ‘Mood’ (p<.001). With respect to age, younger participants reported better scores on ‘body image and eating behavior’ (*p*=.005). With respect to differences due to family structure and glucose monitoring methods, no differences were found. However, regarding therapy, differences were found in social impact (multiple daily injections, MDI (*n* = 19): 77.63 vs. CSII (*n* = 147): 69.84, *p*=.004).

Concurrent validity was assessed by relating the dimensions of the MY-Q to the PedQL 3.0-diabetes module. MY-Q was globally correlated with PedsQL 3.0, global, and with all dimensions in PedsQL 3.0. Similarly, all the MY-Q factors were correlated with all the PedsQL dimensions, except ‘Treatment satisfaction’ of the MY-Q and ‘Worry’ of the PedsQL (Table 6).

Furthermore, a negative correlation was found between Hb1Ac and the global MY-Q and with the factors ‘Parents’, ‘Diabetes Control Perceptions’, ‘Responsibilities’, ‘Worries’, ‘Treatment satisfaction’ and ‘Mood’ (Table 6).

**Table 5:** Levels of the MY-Q for the general sample and the subgroups under study

		MY-Q Total		Social Impact (F1)		Parents (F2)		Diabetes Control Perceptions (F3)			Responsibilities (F4)		Worries (F5)		Treatment satisfaction (F6)		Body image and eating behavior (F7)		Stigma (F8)		Mood (F9)	
		Mean	SD	Mean	SD	Mean	SD	Mean	SD		Mean	SD	Mean	SD	Mean	SD	Mean	SD	Mean	SD	Mean	SD
*Total sample*		66.20	11.12	70.73	12.23	67.69	19.24	71.76		16.27	74.69	20.95	74.69	21.22	83.50	19.51	77.52	16.72	63.70	24.92	57.73	22.80
*Gender* ^*†*^	*Girls*	62.36	10.74	68.79	12.51	63.56	19.85	67.72		16.44	72.44	21.00	70.91	22.40	82.27	19.32	73.97	17.71	59.18	23.58	51.30	21.99
	*Boys*	70.35^*^	10.15	73.75^*^	11.27	74.19*	16.25	77.79*		14.23	78.35*	20.47	80.22*	18.35	85.82	19.44	83.02*	13.49	70.77*	25.30	67.58*	20.38
*Age*	*< 12*	64.87	10.49	71.87	10.40	74.82	17.26	77.60		16.47	70.83	20.07	81.77	13.78	85.93	23.40	87.23	12.29	65.62	25.09	64.66	21.54
	*13–15*	60.74	10.69	68.75	12.72	66.55	20.66	71.71		17.60	72.20	21.17	75.32	22.91	81.08	21.26	77.05	17.68	*64.03*	24.95	53.84	24.53
	*> 16*	61.36	9.89	72.47	12.14	66.34	18.01	69.61		14.31	78.65	20.57	71.34	21.16	85.57	15.50	74.32^*^	15.90	62.05	24.91	59.56	20.67
*Family* *Struc-ture*	*P*	66.65	10.45	71.87	10.90	69.64	17.79	73.31		17.13	76.78	18.50	75.84	21.38	83.40	20.65	79.04	16.26	64.00	23.64	60.40	21.30
	*AP*	64.21	13.27	68.20	16.31	62.80	22.22	68.98		14.85	68.98	26.25	75.46	18.82	85.64	16.87	74.30	17.70	69.13	26.93	54.66	25.54
	*M*	62.71	11.90	68.33	13.27	66.38	21.62	67.50		9.20	71.66	27.33	68.33	24.02	87.50	9.44	72.91	18.84	57.77	30.77	48.80	26.62
	*F*	43.18	.	44.44	.	41.66	.	75.00		.	75.00	.	37.50	.	50.00	.	62.50	.	33.33	.	24.00	.
	*Other*	55.68	8.46	69.44	7.52	54.16	24.53	59.37		15.72	62.50	22.82	68.75	16.13	65.62	18.75	77.52	16.72	47.91	19.69	41.00	16.12
*Glucose monitor-ing method*	*FBGM*	61.37	9.77	75.92	70.41	67.36	20.31	70.83		6.45	56.25	20.53	79.16	12.90	93.75	6.84	73.95	13.92	61.11	33.19	68.00	6.19
	*CGM*	61.58	10.44	70.41	12.17	67.84	18.84	71.82		16.68	75.27	20.84	74.45	21.61	83.69	18.90	77.44	15.59	63.04	25.02	57.18	22.39
	*Both*	62.14	10.60	71.33	12.23	66.85	22.21	71.59		15.99	76.13	20.37	75.00	21.12	79.54	24.56	78.97	23.66	68.56	22.40	58.36	27.78
*Treat-ment*	*CSII*	61.30	10.52	69.84	12.26	67.14	19.22	71.85		16.14	73.80	20.91	74.40	21.31	83.67	19.93	77.42	17.17	63.49	24.89	57.22	23.10
	*MDI*	64.28	9.04	77.63*	9.77	71.92	19.3	71.05		17.70	81.57	20.56	76.97	20.94	82.23	16.30	78.28	13.07	65.35	25.79	61.68	20.49

Notes: P = Living with both parents; AP: Alternately living with parents; M: Living with mother; F: Living with father; FBGM: Fingerstick for blood glucose measurement; CGM: Continuous glucose measurement; CSII: Continuous subcutaneous insulin infusion; MDI: Multiple daily injections

^*^: *p* < .05

^**†**^: One participant identified as another gender and was therefore not included in the analysis

**Table 6:** Correlations among the MY-Q dimensions, PedsQL scores and Hb1Ac scores

	MY-Q Total	F1 Social Impact	F2 Parents	F3 Diabetes Control Perceptions	F4 Respons-ibilities	F5 Worries	F6 Treatment satisfaction	F7 Body image and eating behavior	F8 Stigma	F9 Mood
Hb1Ac	− 0.258**	− 0.065	− 0.258**	− 0.470**	− 0.185*	− 0.199*	− 0.237**	− 0.038	− 0.048	− 0.333**
General QoL	0.567**	0.369**	0.579**	0.369**	0.368**	0.299**	0.179*	0.349**	0.330**	0.484**
PedsQLProblems	0.558**	0.459**	0.466**	0.470**	0.351**	0.394**	0.107	0.161*	0.308**	0.543**
PedsQLTreatment1	0.671**	0.442**	0.686**	0.377**	0.477**	0.365**	0.171*	0.246**	0.486**	0.404**
PedsQLTreatment2	0.499**	0.478**	0.398**	0.359**	0.268**	0.258**	0.198*	0.240**	0.327**	0.299**
PedsQLWorry	0.528**	0.355**	0.339**	0.435**	0.219**	0.697**	0.128	0.201**	0.226**	0.328**
PedsQLComun	0.588**	0.431**	0.473**	0.416**	0.339**	0.306**	0.298**	0.304**	0.397**	0.403**
PedsQLTotal	0.737**	0.579**	0.614**	0.550**	0.437**	0.505**	0.215**	0.281**	0.445**	0.555**

* *p*< .05 ** *p*< .001

Notes: HBA1c= hemoglobin A1c; PedsQL= Pediatric Quality of Life Inventor

## Discussion

The Swedish version of the MY-Q has been linguistically and psychometrically validated to measure health-related quality of life in children and adolescents with T1D aged 10–19 years. The Swedish version of the MY-Q, including new stigma items, presents adequate psychometric properties and conceptual and semantic equivalence with the original Dutch version. While both the 5-factor and 9-factor structures were evaluated, our 2nd-order CFA results indicated that the 9-factor model (original Dutch version) provided a superior fit for the Swedish population (CFI = 0.90, RMSEA = 0.05). Beyond the statistical indices, the 9-factor solution offers greater clinical granularity. By maintaining separate subscales for domains such as ‘Parents’ and ‘Diabetes Control Perceptions’, the MY-Q allows healthcare providers to pinpoint specific areas of distress. This precision is essential for the tool’s primary goal: to facilitate targeted discussions and personalized interventions during brief clinical consultations, which might be obscured in a more condensed 5-factor model.

The high correlation between MY-Q and PedsQL subscales confirms that they measure similar constructs. However, the clinical superiority of one over the other lies in its utility and feasibility. The MY-Q is specifically designed to be brief (max 15 min) and provides direct screening for clinical conditions that warrant immediate attention, such as depressive symptoms and problematic eating. This ensures that the screening is not just a burden of data collection, but a relevant starting point for a person-centered conversation.

In this study, the gender distribution was skewed, with a greater representation of girls. In Sweden, the gender distributions of children and adolescents with T1D are equal [2], and no similar patterns were found either in the psychometric evaluations of the MY-Q [12, 13] or in similar studies in Sweden [9, 22, 23]. However, in the psychometric evaluation of the PedsQL 3.0-diabetes module, more boys were represented [10]. Recruitment to the study was consecutive, and no instructions were initially given that there should be equal gender distribution at recruitment, which may indicate that girls were more willing to participate. However, this finding contradicts the results of the study by Sand et al., which included more boys than girls [10].

From a gender perspective, it should be emphasized that girls with T1D have more unsatisfactory glycemic control than boys do [2, 24]. The significant gender differences observed, with girls reporting lower HRQoL and mood scores across nearly all factors, raise a critical clinical question: do these results reflect a different perception of the questionnaire or a higher underlying burden of the disease? In line with Swedish national reports on adolescent mental health [25, 26], our findings suggest that girls with T1D may face a higher true burden related to social impact, worries, and body image. Rather than comparing girls to a male ‘norm’, these scores should be interpreted in the clinic as indicators of specific psychosocial needs. Clinically, a low score in a female patient should trigger a gender-sensitive, person-centered discussion to identify if she is experiencing higher levels of stigma or distress, ensuring that support is tailored to these distinct challenges.

In the Swedish context, the observed gender gap in mood and HRQoL may be explained by higher levels of societal pressure and stress reported by adolescent girls nationally, which likely exacerbates the perceived burden of diabetes management. In addition, several similar studies on young people with T1D have shown that girls have a lower HRQoL than boys do, which correlates with glycemic outcomes [10, 22, 26]. This emphasizes the importance of measuring HRQoL to provide person-centered support.

MY-Q is a questionnaire developed for use in clinical settings that could be clinically useful. To support interpretation and clinical discussion with the adolescent, a flag is generated when one of the two most adverse Likert-scale responses is selected for any item within the domain [12]. In the Dutch context, a project was carried out to measure health-related QoL via the MY-Q in routine care, which was perceived as useful. However, most clinics were unable to continue after the project due to logistics issues such as a lack of time and shortages of healthcare providers [27]. Despite this, there is ambition to prioritize the integration of PROs into regular care in Sweden, as stated in the ISPAD guidelines [5].

In the current study, the treatment satisfaction scores were high. A partial explanation for this may be that in Sweden, persons with T1D can choose the technical diabetes equipment that suits them best and does not need to take the cost into account, since all equipment for both insulin dosing and glucose measurement are reimbursed and free of charge. Treatment satisfaction was negatively correlated with HbA1c and positively correlated with HRQoL, which was also shown in another study among young people in Sweden [28]. Our study revealed that girls perceived more issues than boys did, which is equivalent to the results presented in a study by Ortiz-Domenech et al. [29].

Health-related stigma is defined as a personal experience characterized by exclusion, rejection, or blame, resulting in adverse social judgment about a person with a specific health condition [30]. In 2017, a study on diabetes stigma was carried out using unvalidated questions in 5,422 people with diabetes (96% adults). Among those with T1D, 76% perceived diabetes-related stigma, and an association between diabetes stigma and technology use was observed [31]. In our study, there was no relationship between stigma and Hb1Ac. This finding seems to correlate with findings in other studies, in which glycemic control was better explained by the relationship between treatment satisfaction and the perception of self-efficacy [32, 33], which, in our study, was related to HbA1c. In this study, both social impact and stigma are variables that can help healthcare professionals identify problems in other areas that have a direct impact on clinical outcomes and QoL. The inclusion of the stigma factor in the MY-Q questionnaire is clearly important, as addressing stigma may be an avenue to emotionally support young people with T1D.

The recruitment challenges observed in this study, primarily linked to the high workload and limited resources of the participating pediatric clinics, highlight the barriers to implementing PROMs in routine care. To address potential ‘questionnaire fatigue’ and improve clinical feasibility, future research should explore the development of a shortened version of the MY-Q or a two-step screening approach. In such a model, a single ‘sentinel item’ from each subscale could be used as an initial screen, with the full subscale only administered if the initial score indicates a risk. Regarding the frequency of administration, incorporating the MY-Q into the annual review (as currently practiced in Dutch implementation studies) may provide a sustainable balance between longitudinal monitoring and clinical burden.

When comparing the MY-Q with other translated measures available in Sweden, its main clinical advantage lies in its design, which prioritizes practical outcomes over purely descriptive data. The implementation of these tools in busy pediatric clinics often fails not due to a lack of validity, but rather because of the logistical burden and fatigue generated by the questionnaires. Therefore, prospects for successful implementation in the Swedish context should focus on digital integration into the National Diabetes Registry (NDR) or the Electronic Health Record. By using the MY-Q within a tiered care approach (where it functions as an annual psychosocial check-up), clinics can provide structured, person-centered support without overburdening limited staff resources. This approach ensures that psychosocial follow-up becomes a sustainable part of routine clinical practice, rather than an isolated research effort.

### Methodological discussion

In this study, four translators who speak Swedish and Dutch fluently were used. In addition, all the authors were involved in the entire linguistic process of the questionnaire. When considering questions about stigma, we included only three questions. This approach is insufficient for measuring stigma with only three items, but it can, in a clinical context, be a way to identify potential stigma-related issues and further address them in consultation.

A strength of this study is that we included participants from nine pediatric clinics in different regions of Sweden. Furthermore, the clinics were of different sizes and represented young people from rural areas to large cities. We chose to include participants consecutively, which resulted in more girls than boys answering the questionnaires. The recruitment and data collection were managed mainly by nurses at the participating clinics, and we did not obtain any information about how many declined to participate. Most (seven) of the clinics were small (< 160 patients) [2], heavily burdened, and had insufficient resources, which resulted in difficulties in prioritizing data collection for this study. Various reminders and encouragement were sent to the clinics. Despite this, the data collection took longer than planned. However, this should not have negatively affected our results. One strength is that data collection took place over the entire year, which may have resulted in a more representative sample.

Cronbach’s alpha and McDonald’s omega were calculated for internal consistency estimates. McDonald’s omega considers factor loadings, making it a more accurate and reliable indicator than Cronbach’s alpha is [21]. We have chosen to present both, as Cronbach’s alpha is well known.

## Conclusions

The linguistic and psychometric evaluation of the MY-Q has shown that the Swedish My-Q presents adequate psychometric properties and conceptual and semantic equivalence with the original Dutch version. The MY-Q provides a valuable culturally adapted option for the Swedish context, uniquely combining traditional HRQoL measurement with stigma assessment. Its design makes it a practical tool for integrating psychosocial monitoring into routine pediatric diabetes care. Furthermore, there are no questionnaires that measure stigma in young people with T1D. This psychometric evaluation revealed that the addition of these items was effective and could be helpful in the clinical use of the MY-Q. However, future research should focus on developing a questionnaire to measure stigma and evaluate the implementation of the My-Q in a clinical context.

## Supplementary Information

Below is the link to the electronic supplementary material.

Supplementary Material 1

## Data Availability

The datasets used and analyzed during the current study are available from the corresponding author upon reasonable request.

## Abbreviations

CFA: Confirmatory Factor Analysis
CFI: Comparative Fit Index
CGM: Continuous Glucose Measuring
CSII: Continuous Subcutaneous Insulin Infusion
DSAS-1: Diabetes Stigma Assessment Scale
HbA1c: Glycated Haemoglobin
HRQoL: Health Related Quality of Life
MY-Q: the MIND Youth Questionnaire into Swedish
PedsQL: Pediatric Quality of Life Inventory
PRO: Patient Reported Outcomes
PROM: Patient Reported Outcomes Measure
T1D: Type 1 Diabetes
QoL: Quality of Life
RMSEA: Root Mean Square Error of Approximation
SRMR: Standardized Root Mean Square Residual
WLSMV: Weighted Least Square Mean and Variance
